# Supplementary material for: Rapid age-grading and species identification of natural mosquitoes for malaria surveillance
Source: Nat Commun. 2022 Mar 21;13:1501. doi: 10.1038/s41467-022-28980-8 (PMC8938457; doi:10.1038/s41467-022-28980-8)
Supplement: Supplementary file 1 — Supplementary Information [file 41467_2022_28980_MOESM1_ESM.pdf]

Rapid age-grading and species identification of natural mosquitoes for malaria surveillance

Doreen J. Siria[1,*], Roger Sanou[2,*], Joshua Mitton[3, 4, 5, *], Emmanuel P. Mwanga[1, 3], Abdoulaye Niang[2], Issiaka Sare[2], Paul C.D. Johnson[3], Geraldine M. Foster[6], Adrien M.G. Belem[7], Klaas Wynne[4], Roderick Murray-Smith[5], Heather M. Ferguson[1, 3], Mario González-Jiménez[4, §], Simon A. Babayan[3, §], Abdoulaye Diabaté[2,†], Fredros O. Okumu[1, 3,†], and Francesco Baldini[3,†, §]

[1]Environmental Health & Ecological Sciences Department, Ifakara Health Institute, Off Mlabani Passage, PO Box 53, Ifakara, Tanzania
[2]Institut de Recherche en Sciences de la Santé (IRSS)/Centre Muraz, Bobo-Dioulasso, Burkina Faso
[3]Institute of Biodiversity Animal Health and Comparative Medicine, University of Glasgow, Glasgow G12 8QQ, UK
[4]School of Chemistry, University of Glasgow, Glasgow G12 8QQ, UK
[5]School of Computing Science, University of Glasgow, Glasgow, G12 8QQ, UK
[6]Department of Vector Biology, Liverpool School of Tropical Medicine, Liverpool, L3 5QA, UK
[7]Université Nazi Boni de Bobo-Dioulasso, Bobo-Dioulasso, PO 1091, Burkina Faso
[*]These authors contributed equally to this work.
[†]These authors equally supervised this work.
[§]Corresponding authors: Mario.GonzalezJimenez@glasgow.ac.uk, Simon.Babayan@glasgow.ac.uk, Francesco.Baldini@glasgow.ac.uk

Supplementary information

Supplementary Tables

Location	Species	Strain	Reference
Glasgow, UK	*An. gambiae*	Kisumu	[1]
	An. coluzzii	Ngousso	[2]
	An. arabiensis	Ifakara	[3]
Ifakara, TZ	*An. gambiae*	Ifakara, Niage	[4]
	An. arabiensis	Ifakara, Niage	[5]
Bobo-Dioulasso, BF	*An. gambiae*	Soumousso	[6]
	An. coluzzii	Vallée du Kou	[6]

Supplementary Table 1 List of species and strains used for training DL-MIRS.

Type of data	Species	Burkina Faso	Tanzania	United Kingdom	Discarded spectra
LV	*An. gambiae*	2005	8604	2277	1416
	An. coluzzii	2228	0	2491	
	An. arabiensis	0	8557	1661	
GV	*An. gambiae*	2904	0	0	640
	An. coluzzii	1027	0	0	
	An. arabiensis	0	5687	0	
EV	*An. gambiae*	265	1418	0	244
	An. coluzzii	248	0	0	
	An. arabiensis	0	1346	0	
Wild Dissected	*An. coluzzii*	335	0	0	11
	An. arabiensis	0	758	0	
Wild Non-dissected	*An. coluzzii*	568	0	0	0
	An. arabiensis	0	834	0	

Supplementary Table 2 Sample sizes per origin and species. Source data are provided as TableS2.csv[7] .

**Study Laboratory Variation (LV)**
Data from group LV only.
8224 mosquito data points.
Data from Tanzania, Burkina Faso, United Kingdom.
Data balanced, where possible, by country, species, and age groups.
**Study Genetic Variation (GV)**
Data from groups LV and GV.
4800 mosquito data points from group LV and 2400 mosquito data points from group GV.
Data from Tanzania and Burkina Faso only.
Data balanced by country, species, and age groups.
Testing data set is a 10% split of group GV data only.
**Study Environmental Variation (EV)**
Implicit use of data from groups LV and GV (7200 data points).
Data from group EV.
A varying number of data points from the EV group.
EV data from Tanzania and Burkina Faso only.
Data balanced by country, species, and age groups for groups LV and GV, and balanced where possible for group EV.
Testing data set is a hold out data set of 180 EV data points not included in the training set.
**Study Wild Populations**
Implicit use of data from groups LV and GV (7200 data points).
Data from wild mosquitoes.
Data balanced by country, species, and age groups for groups LV and GV, and unbalanced for wild group.
In Burkina Faso, the transfer learning with the wild data set is composed by 205 G0, 104 G1, 26 G234 (total 335 data points, 4.4% of the whole training set).
In Tanzania, the transfer learning with the wild data set is composed by 168 G0, 573 G1, 17 G234 (total 758 data points, 9.5% of the whole training set).
Testing data set is wild data set of 568 (Burkina Faso) and 834 (Tanzania) non-dissected mosquito data points not included in the training set.

Supplementary Table 3 Allocation of samples between training and testing of DL-MIRS. Source data are provided as TableS3.csv[7] .

**E1 – Reducting parameters**
Data from groups LV, GV, and EV only.
4800 mosquito data points from group LV and 2400 mosquito data points from group GV in the training set.
Data from Tanzania and Burkina Faso only.
Data balanced by country, species, and age groups for groups LV and GV.
Testing data set is group EV.
Consider how the number of trainable parameters in the model affects classification accuracy.
**E2 – Cross EV**
Data from groups GV, and EV.
2400 mosquito data points from group GV in the training set and either 1200 data points from Tanzania or 300 data points from Burkina Faso from Group EV.
Data from Tanzania and Burkina Faso only.
Data balanced by country, species, and age groups for groups GV. Data balanced by species, and age groups for groups EV.
Testing data set is data from the opposite country to that which was included during training from group EV.
Attempt to classify unseen EV data from another country.
**E3 - Cross laboratory**
Data from group LV only.
8224 mosquito data points.
Data balanced, where possible, by country, species, and age groups.
Leave data out from one country as the testing set in an attempt to classify data from an unseen lab.
**E4 – Cross laboratory with reduced parameters**
Data from group LV only.
Stage 1:
Initial training set is 3424 mosquito data points from UK group LV, balanced where possible by species, and age groups.
Sensitivity analysis performed on model from stage 1 to select frequencies.
Stage 2:
Training set is 2400 mosquito data points from either Tanzania or Burkina Faso in group LV, balanced by species, and age groups.
Testing data set is from group LV from the country left out during training.

Supplementary Table 4 Grouping of mosquito subsets for cross-validation and generalisation of DL-MIRS.

Supplementary Table 5 Predicted power to detect a shift in age structure in response to each of two interventions, long-lasting insecticide-treated nets (LLIN) and attractive toxic sugar baits (ATSB) relative to a population with no intervention (see Fig. 5). Power depended on the number of mosquitoes sampled from each population (intervention and control) and the number of spectra from semi-field (EV) mosquitoes used in the training set. Each power value was estimated from analysis of 10,000 simulated data sets. Source data are provided as TableS5.csv[7] .

N sampled	N EV spectra added	Power	
		LLIN intervention	ATSB intervention
20	0	6.9%	5.0%
20	162	46.1%	14.8%
20	324	60.0%	18.6%
20	486	60.9%	18.8%
20	654	74.6%	22.7%
20	815	78.0%	24.4%
20	973	75.0%	23.4%
20	1131	79.0%	24.3%
20	1294	80.7%	25.8%
20	1452	78.5%	24.9%
50	0	10.3%	6.4%
50	162	84.6%	31.1%
50	324	94.9%	40.5%
50	486	95.2%	41.7%
50	654	98.8%	50.5%
50	815	99.3%	53.8%
50	973	98.9%	51.2%
50	1131	99.4%	54.3%
50	1294	99.4%	55.2%
50	1452	99.2%	54.4%
100	0	15.4%	7.5%
100	162	99.1%	55.8%
100	324	99.9%	69.5%
100	486	99.9%	70.0%
100	654	100.0%	80.5%
100	815	100.0%	83.2%
100	973	100.0%	80.8%
100	1131	100.0%	82.9%
100	1294	100.0%	84.7%
100	1452	100.0%	83.2%
150	0	20.6%	8.7%
150	162	99.9%	73.5%
150	324	100.0%	85.6%
150	486	100.0%	86.4%
150	654	100.0%	93.6%
150	815	100.0%	94.7%
150	973	100.0%	93.3%
150	1131	100.0%	94.7%
150	1294	100.0%	95.4%
150	1452	100.0%	95.4%
200	0	25.9%	10.6%
200	162	100.0%	84.1%

Continued on next page...

Table 5 continued from previous page

N sampled	N EV spectra added	Power	
		LLIN intervention	ATSB intervention
200	324	100.0%	93.9%
200	486	100.0%	94.6%
200	654	100.0%	97.8%
200	815	100.0%	98.7%
200	973	100.0%	98.2%
200	1131	100.0%	98.3%
200	1294	100.0%	98.7%
200	1452	100.0%	98.6%
250	0	30.9%	11.6%
250	162	100.0%	91.9%
250	324	100.0%	97.6%
250	486	100.0%	97.3%
250	654	100.0%	99.4%
250	815	100.0%	99.7%
250	973	100.0%	99.5%
250	1131	100.0%	99.6%
250	1294	100.0%	99.7%
250	1452	100.0%	99.6%
300	0	36.5%	13.4%
300	162	100.0%	95.3%
300	324	100.0%	99.1%
300	486	100.0%	99.2%
300	654	100.0%	99.7%
300	815	100.0%	100.0%
300	973	100.0%	99.8%
300	1131	100.0%	100.0%
300	1294	100.0%	100.0%
300	1452	100.0%	99.9%

Supplementary Table 6 List of wild samples collected in the villages of Vallée du Kou 5 (VK5) in Burkina Faso (BF) and Sululu in Tanzania (TZ). *An. coluzzii* or *An. arabiensis* were collected from the field and dissected either on the same day or 2-3 days after collection, and their gonotrophic cycle determined based on ovarian characterisation. Female that were undergoing or completed egg development (oogenesis) at the time of dissection could not be assigned to a gonotrophic cycle. For each collection, a number of non-dissected mosquitoes that was equal to those dissected on each day was preserved for subsequent DL-MIRS analysis. Source data are provided as TableS6.csv[7] .

Location	Species	Collected	Dissected	Gonotrophic cycle						Not dissected
				0	1	2	3	4	Oogenesis	
VK5 - BF	*An. coluzzii*	15/05/2021	15/05/2021	4	1	1	0	0	4	10
VK5 - BF	*An. coluzzii*	19/05/2021	19/05/2021	5	0	0	0	0	0	5
VK5 - BF	*An. coluzzii*	23/05/2021	25/05/2021	4	0	1	0	0	0	5
VK5 - BF	*An. coluzzii*	27/05/2021	08/06/2021	12	0	0	0	0	0	12
VK5 - BF	*An. coluzzii*	31/05/2021	21/06/2021	4	2	1	0	0	5	12
VK5 - BF	*An. coluzzii*	04/06/2021	14/07/2021	9	0	0	0	0	0	9
VK5 - BF	*An. coluzzii*	08/06/2021	17/05/2021	2	1	0	0	0	14	16
VK5 - BF	*An. coluzzii*	12/06/2021	21/05/2021	5	1	1	1	0	16	24
VK5 - BF	*An. coluzzii*	16/06/2021	27/05/2021	6	2	0	0	0	15	24
VK5 - BF	*An. coluzzii*	20/06/2021	10/06/2021	28	12	1	0	0	6	50
VK5 - BF	*An. coluzzii*	24/06/2021	23/06/2021	1	15	2	1	0	81	100
VK5 - BF	*An. coluzzii*	28/06/2021	16/07/2021	43	3	0	0	0	6	52
VK5 - BF	*An. coluzzii*	02/07/2021	18/05/2021	2	0	1	0	0	10	13
VK5 - BF	*An. coluzzii*	06/07/2021	22/05/2021	0	0	0	0	0	6	6
VK5 - BF	*An. coluzzii*	10/07/2021	28/05/2021	0	1	0	1	0	18	21
VK5 - BF	*An. coluzzii*	14/07/2021	11/06/2021	14	11	4	1	0	0	30
VK5 - BF	*An. coluzzii*	18/07/2021	24/06/2021	17	27	6	3	0	27	80
VK5 - BF	*An. coluzzii*	22/07/2021	17/07/2021	49	26	2	0	0	19	96
Total VK5	*An. coluzzii*	-	-	205	102	20	7	0	227	565
Sululu - Tz	*An. arabiensis*	04/05/2021	04/05/2021	15	14	1	0	0	0	30
Sululu - Tz	*An. arabiensis*	07/05/2021	07/05/2021	2	14	0	0	0	9	25
Sululu - Tz	*An. arabiensis*	11/05/2021	11/05/2021	4	20	0	0	0	1	25
Sululu - Tz	*An. arabiensis*	18/05/2021	18/05/2021	2	13	0	0	0	3	18
Sululu - Tz	*An. arabiensis*	21/05/2021	21/05/2021	3	20	0	0	0	2	25
Sululu - Tz	*An. arabiensis*	25/05/2021	25/05/2021	6	16	0	0	0	3	25
Sululu - Tz	*An. arabiensis*	29/05/2021	29/05/2021	2	14	0	0	0	4	20
Sululu - Tz	*An. arabiensis*	04/06/2021	04/06/2021	8	15	0	0	0	7	30
Sululu - Tz	*An. arabiensis*	08/06/2021	08/06/2021	7	20	0	0	0	3	30
Sululu - Tz	*An. arabiensis*	15/06/2021	15/06/2021	4	21	2	0	0	0	27
Sululu - Tz	*An. arabiensis*	18/06/2021	18/06/2021	6	15	2	0	0	0	23
Sululu - Tz	*An. arabiensis*	22/06/2021	22/06/2021	3	27	2	0	0	3	35
Sululu - Tz	*An. arabiensis*	25/06/2021	25/06/2021	6	15	0	0	0	3	24
Sululu - Tz	*An. arabiensis*	29/06/2021	29/06/2021	15	8	0	0	0	0	23
Sululu - Tz	*An. arabiensis*	02/07/2021	02/07/2021	10	10	0	0	0	0	20
Sululu - Tz	*An. arabiensis*	06/07/2021	06/07/2021	6	9	0	0	0	0	15
Sululu - Tz	*An. arabiensis*	13/07/2021	13/07/2021	4	7	0	0	0	0	11
Sululu - Tz	*An. arabiensis*	16/07/2021	16/07/2021	4	9	1	1	0	0	15
Sululu - Tz	*An. arabiensis*	20/07/2021	20/07/2021	5	3	0	0	0	1	9
Sululu - Tz	*An. arabiensis*	23/07/2021	23/07/2021	7	1	0	0	0	8	16
Sululu - Tz	*An. arabiensis*	30/07/2021	30/07/2021	1	5	0	1	0	1	8
Sululu - Tz	*An. arabiensis*	03/08/2021	03/08/2021	1	6	0	0	0	0	7

Table 6 continued from previous page

Location	Species	Collected	Dissected	Gonotrophic cycle 0	1	2	3	4	Oogenesis	Not dissected
Sululu - Tz	*An. arabiensis*	06/08/2021	06/08/2021	4	5	0	0	0	0	9
Sululu - Tz	*An. arabiensis*	10/08/2021	10/08/2021	10	4	0	0	0	1	15
Sululu - Tz	*An. arabiensis*	13/08/2021	13/08/2021	3	3	0	0	0	11	17
Sululu - Tz	*An. arabiensis*	17/08/2021	17/08/2021	14	6	0	0	0	0	20
Sululu - Tz	*An. arabiensis*	24/08/2021	24/08/2021	8	7	0	0	0	3	18
Sululu - Tz	*An. arabiensis*	31/08/2021	31/08/2021	9	5	0	0	0	0	14
Sululu - Tz	*An. arabiensis*	27/04/2021	29/04/2021	0	21	1	1	0	2	25
Sululu - Tz	*An. arabiensis*	28/04/2021	30/04/2021	0	30	1	0	0	4	35
Sululu - Tz	*An. arabiensis*	29/04/2021	01/05/2021	5	41	1	0	0	3	50
Sululu - Tz	*An. arabiensis*	04/05/2021	06/05/2021	2	21	1	0	0	1	25
Sululu - Tz	*An. arabiensis*	07/05/2021	09/05/2021	1	13	0	0	0	1	15
Sululu - Tz	*An. arabiensis*	11/05/2021	13/05/2021	0	14	0	0	0	1	15
Sululu - Tz	*An. arabiensis*	15/05/2021	17/05/2021	0	14	0	0	0	0	14
Sululu - Tz	*An. arabiensis*	18/05/2021	20/05/2021	2	26	2	0	0	0	30
Sululu - Tz	*An. arabiensis*	21/05/2021	23/05/2021	2	21	1	0	0	1	25
Sululu - Tz	*An. arabiensis*	25/05/2021	27/05/2021	5	18	0	0	0	2	25
Sululu - Tz	*An. arabiensis*	29/05/2021	31/05/2021	1	15	1	0	0	3	20
Sululu - Tz	*An. arabiensis*	04/06/2021	06/06/2021	8	20	0	0	0	3	31
Sululu - Tz	*An. arabiensis*	08/06/2021	10/06/2021	9	14	1	0	0	1	25
Sululu - Tz	*An. arabiensis*	15/06/2021	17/06/2021	6	6	0	0	0	0	12
Sululu - Tz	*An. arabiensis*	18/06/2021	20/06/2021	3	11	0	0	0	0	14
Sululu - Tz	*An. arabiensis*	22/06/2021	24/06/2021	8	18	0	0	0	2	28
Sululu - Tz	*An. arabiensis*	29/06/2021	01/07/2021	10	16	0	0	0	0	26
Sululu - Tz	*An. arabiensis*	02/07/2021	04/07/2021	13	13	0	0	0	1	27
Sululu - Tz	*An. arabiensis*	06/07/2021	08/07/2021	5	12	0	0	0	0	17
Sululu - Tz	*An. arabiensis*	13/07/2021	15/07/2021	3	7	0	0	0	0	10
Sululu - Tz	*An. arabiensis*	16/07/2021	18/07/2021	3	15	2	0	0	0	20
Sululu - Tz	*An. arabiensis*	20/07/2021	22/07/2021	4	2	0	0	0	3	9
Sululu - Tz	*An. arabiensis*	23/07/2021	25/07/2021	3	9	0	0	0	4	16
Sululu - Tz	*An. arabiensis*	30/07/2021	01/08/2021	1	7	0	0	0	0	8
Sululu - Tz	*An. arabiensis*	03/08/2021	05/08/2021	1	2	0	0	0	2	5
Sululu - Tz	*An. arabiensis*	06/08/2021	08/08/2021	2	6	0	0	0	0	8
Sululu - Tz	*An. arabiensis*	10/08/2021	12/08/2021	8	8	0	0	0	0	16
Sululu - Tz	*An. arabiensis*	13/08/2021	15/08/2021	1	12	0	0	0	4	17
Sululu - Tz	*An. arabiensis*	17/08/2021	19/08/2021	3	14	0	0	0	1	18
Sululu - Tz	*An. arabiensis*	24/08/2021	26/08/2021	4	10	0	0	0	4	18
Sululu - Tz	*An. arabiensis*	31/08/2021	02/09/2021	6	4	0	0	0	2	12
Total Sululu	*An. arabiensis*	-	-	288	752	19	3	0	108	1170

Supplementary Figures

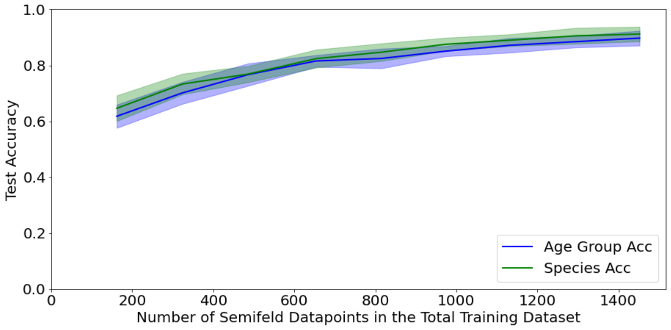

Supplementary Fig. 1 Classification accuracy when training with EV data only. Classification accuracy of up to 90% for age group and 91% for species with a training set comprising up to 1452 semi-field (EV) mosquitoes used to train the model. The solid and shaded lines indicate the mean and standard deviation of the mean of 20 trained models, respectively.

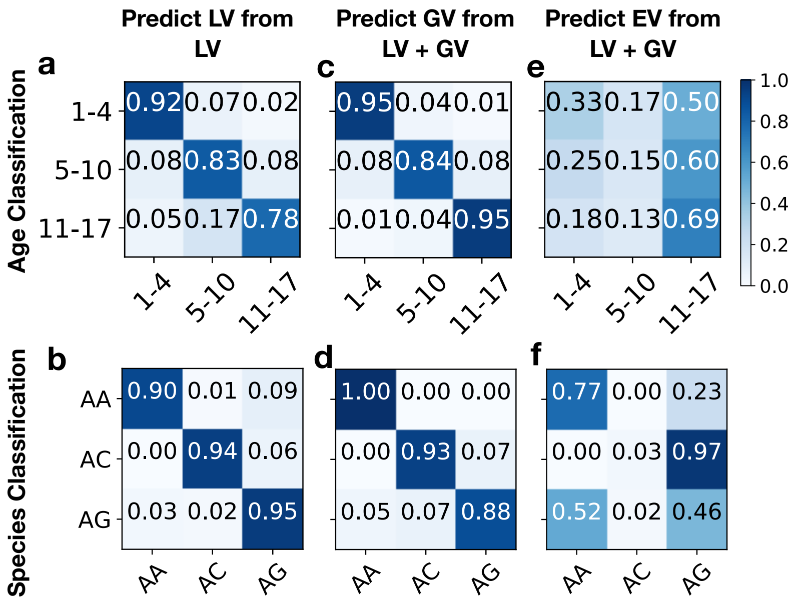

Supplementary Fig. 2 Confusion matrices of model prediction accuracies for models trained on LV and GV mosquitoes only. DL-MIRS was trained using different combinations of mosquitoes from either laboratory larvae reared in the lab (LV, laboratory variation), larvae from the field reared in the lab (GV, genetic variation), or laboratory larvae reared in semi-field (EV, environmental variation). **a—d**, The models were trained on LV or LV+GV mosquitoes (a, b LV alone; or c, d LV+GV) and tested for their ability to classify a random stratified hold-out test set into the correct age class (a, c) or species (b, d). **e**, **f**, We then trained models on LV+GV mosquitoes, and tested their accuracy in correctly identifying EV mosquito ages (e) and species (f).

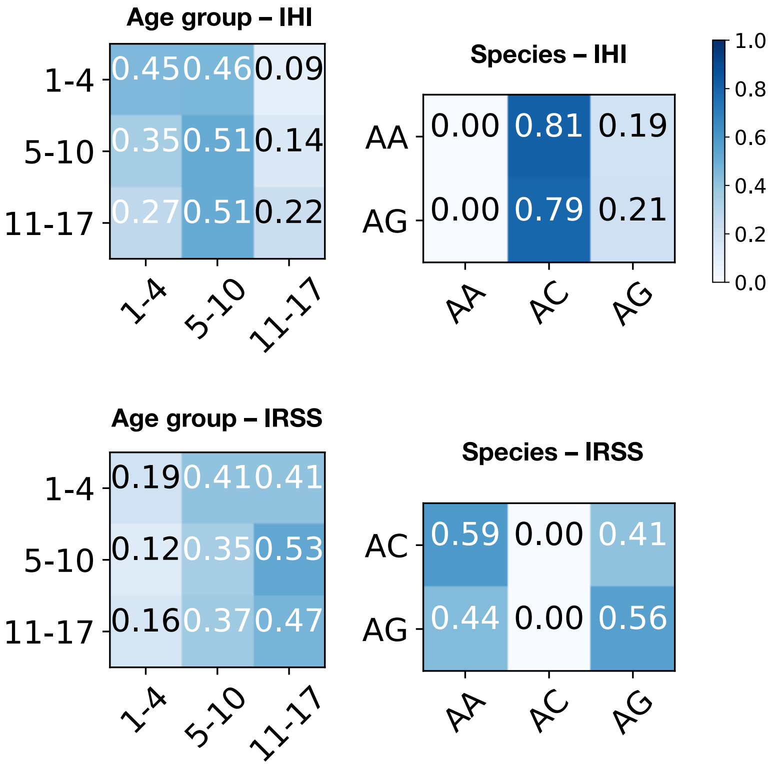

Supplementary Fig. 3 Preselecting wavenumber values from sensitivity analysis of a CNN. A fully connected neural network was then trained, where the input was the wavenumber values identified from the sensitivity analysis on UK data, on IHI and IRSS LV data separately. These models were then tested on LV data from either IHI or IRSS depending which was left out during training. These results demonstrate that no performance increase can be gained by preselecting wavenumber values, consistent with the argument that the deep CNN model is not overfitting when generalisation is poor.

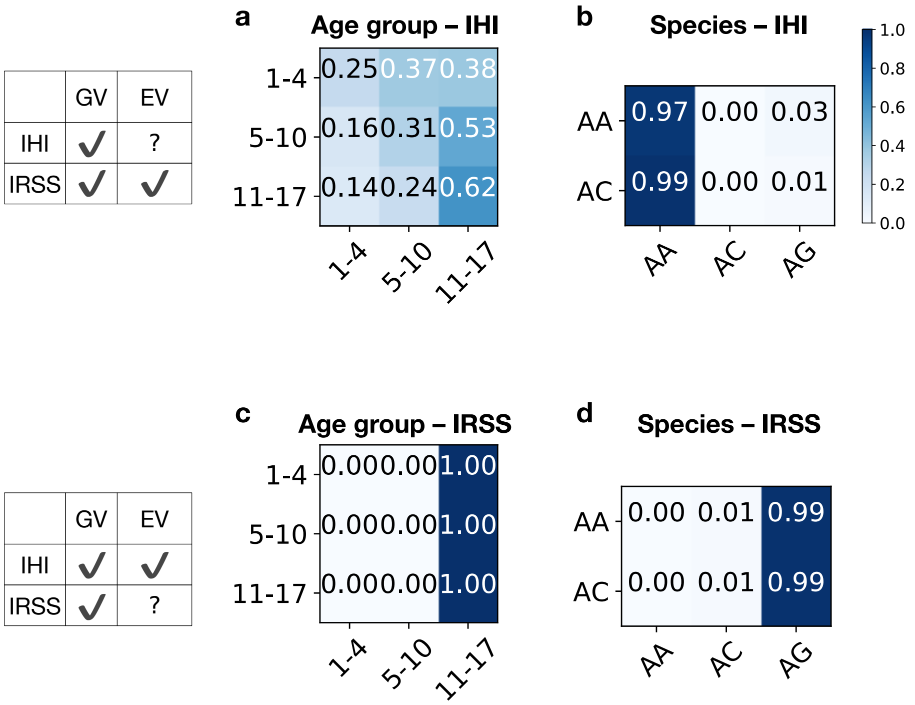

Supplementary Fig. 4 EV data from an unseen site. All of the EV samples from one site were held out and DL-MIRS was trained on the rest of GV and EV samples from IHI (**a**, **b**, age and species, respectively) and IRSS (**c**, **d**, age and species, respectively). This demonstrates that if the training dataset only has GV data for a site it will not be possible for the model to classify EV data, despite the inclusion of EV data from other sites.

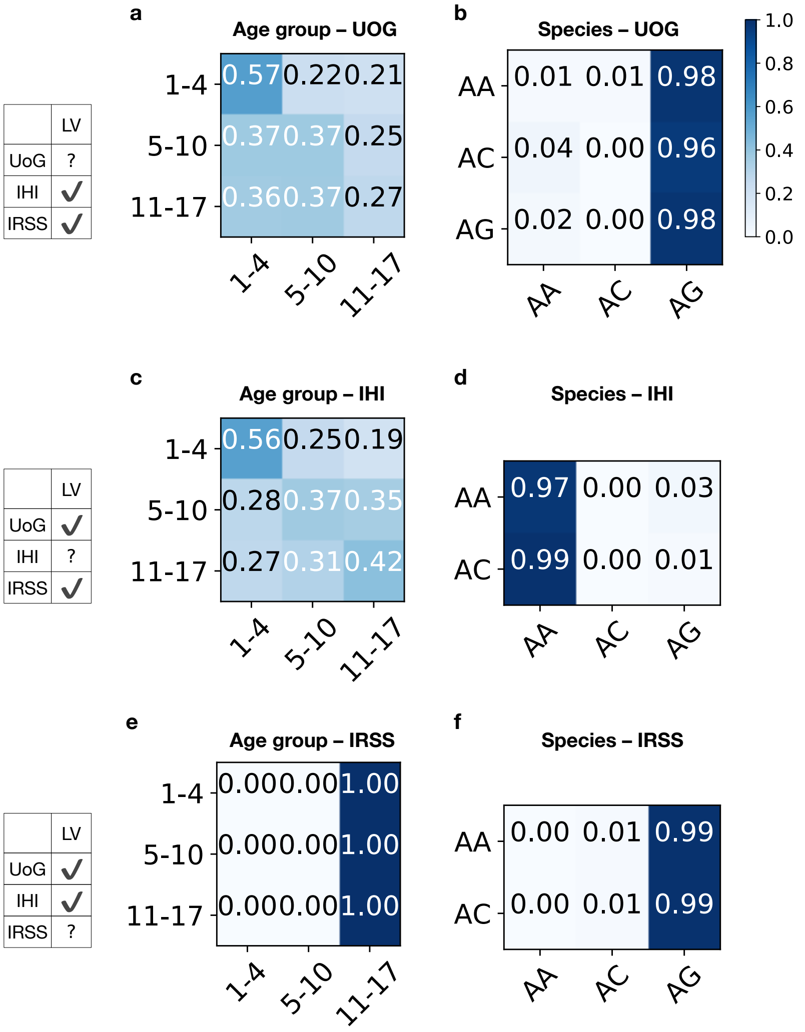

Supplementary Fig. 5 LV data from an unseen site. DL-MIRS was trained with LV data from two sites and then used to classify LV data from a third site (UoG in **a** and **b**, ; IHI in **c** and **d**; IRSS in **e** and **f**). The results demonstrate that the model is not able to learn features capable of classifying data from a previously unseen laboratory. This shows that differences exist in the MIRS data across laboratories from different sites. The result is in agreement with the initial UMAP data exploration, demonstrating differences across laboratories.

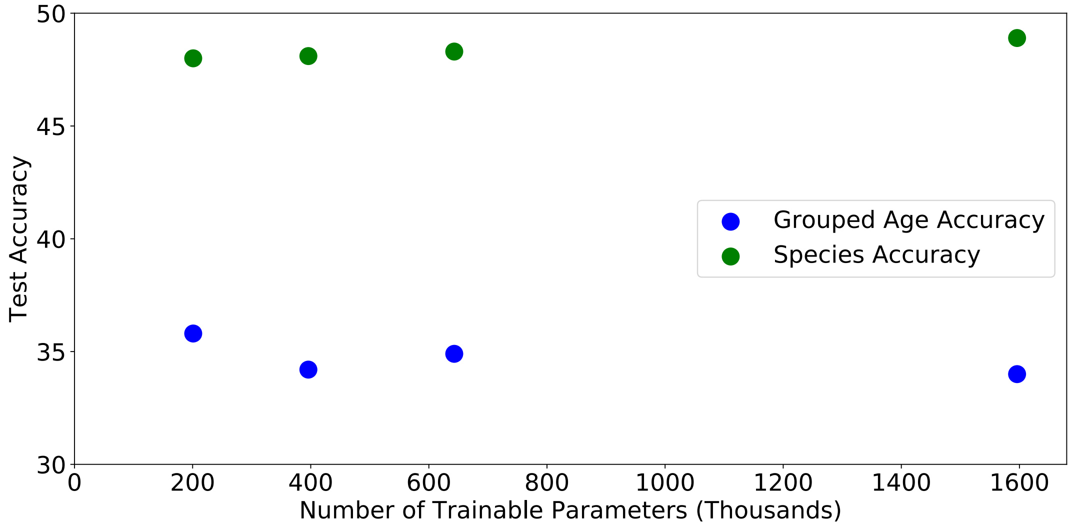

Supplementary Fig. 6 Model overfitting. The CNN was modified to reduce capacity, lowering the number of trainable parameters, to explore the effect on classification accuracy with a testing dataset comprising of EV data only, while the training dataset comprises of LV and GV data. This suggests that the model is not overfitting in the case of training on LV and GV data only, as the model does not increase testing accuracy on EV data when lowering the number of trainable parameters.

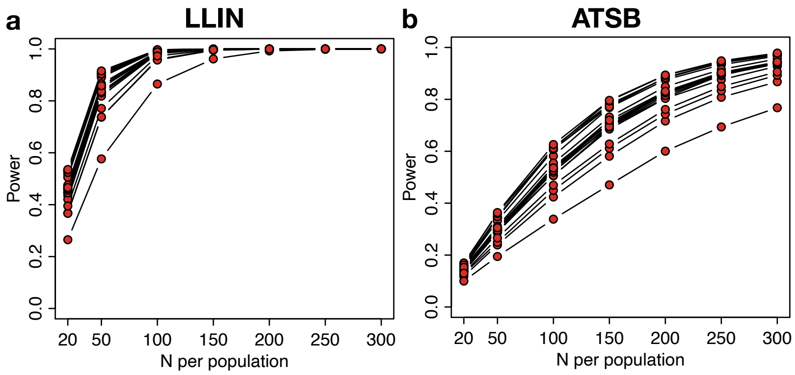

Supplementary Fig. 7 Impact of the number of mosquitoes used for DL-MIRS training on power to detect an effect of vector control on mosquito population age structure for each of two vector control interventions, **a**, long-lasting insecticide-treated nets (LLIN) and **b**, attractive toxic sugar baits (ATSB) relative to a population with no intervention (control).

Supplementary References

1. MR4. *BEI Resources Knowledge Base* `https://www.beiresources.org`. 2021.
2. Nsango, S. E. *et al.* Genetic clonality of *Plasmodium falciparum* affects the outcome of infection in Anopheles gambiae. *Int. J. Parasitol.* **42,** 589–595 (2012).
3. Lyimo, I. *et al.* The impact of host species and vector control measures on the fitness of African malaria vectors. *Proc Biol Sci* **280,** 20122823 (2013).
4. Ng'habi, K. *et al.* Effect of larval crowding on mating competitiveness of *Anopheles gambiae* mosquitoes. *Malar J* **4,** 49 (2005).
5. Ng'habi, K., Mwasheshi, D., Knols, B. & Ferguson, H. Establishment of a self-propagating population of the African malaria vector *Anopheles arabiensis* under semi-field conditions. *Malar J* **9,** 356 (2010).
6. Bilgo, E. *et al.* Transgenic *Metarhizium pingshaense* synergistically ameliorates pyrethroid-resistance in wild-caught, malaria-vector mosquitoes. *PLoS One* **13,** e0203529 (2018).
7. Babayan, S. *SimonAB/DL-MIRS_Siria_et_al: Public release* version v1.0.0. Feb. 2022. `https://doi.org/10.5281/zenodo.5996316`.
