# Supplementary material for: Rapid age-grading and species identification of natural mosquitoes for malaria surveillance
Source: Nat Commun. 2022 Mar 21;13:1501. doi: 10.1038/s41467-022-28980-8 (PMC8938457; doi:10.1038/s41467-022-28980-8)
Supplement: Supplementary file 3 — Reporting Summary [file 41467_2022_28980_MOESM3_ESM.pdf]

nature portfolio

Corresponding author(s): Francesco Baldini

Last updated by author(s): 9th February 2022

Reporting Summary

Nature Portfolio wishes to improve the reproducibility of the work that we publish. This form provides structure for consistency and transparency in reporting. For further information on Nature Portfolio policies, see our Editorial Policies and the Editorial Policy Checklist.

Statistics

For all statistical analyses, confirm that the following items are present in the figure legend, table legend, main text, or Methods section.

n/a	Confirmed	
☐	☒	The exact sample size (*n*) for each experimental group/condition, given as a discrete number and unit of measurement
☐	☒	A statement on whether measurements were taken from distinct samples or whether the same sample was measured repeatedly
☐	☒	The statistical test(s) used AND whether they are one- or two-sided<br>*Only common tests should be described solely by name; describe more complex techniques in the Methods section.*
☐	☒	A description of all covariates tested
☐	☒	A description of any assumptions or corrections, such as tests of normality and adjustment for multiple comparisons
☐	☒	A full description of the statistical parameters including central tendency (e.g. means) or other basic estimates (e.g. regression coefficient) AND variation (e.g. standard deviation) or associated estimates of uncertainty (e.g. confidence intervals)
☐	☒	For null hypothesis testing, the test statistic (e.g. *F*, *t*, *r*) with confidence intervals, effect sizes, degrees of freedom and *P* value noted<br>*Give P values as exact values whenever suitable.*
☒	☐	For Bayesian analysis, information on the choice of priors and Markov chain Monte Carlo settings
☒	☐	For hierarchical and complex designs, identification of the appropriate level for tests and full reporting of outcomes
☒	☐	Estimates of effect sizes (e.g. Cohen's *d*, Pearson's *r*), indicating how they were calculated

Our web collection on statistics for biologists contains articles on many of the points above.

Software and code

Policy information about availability of computer code

Data collection	Mid-infrared spectra were obtained using Bruker Vertex 70 spectrometer or FTIR spectrometer using Bruker OPUS Spectroscopy Software.
Data analysis	Machine learning was performed in Python v.3.6.8 using scikit-optimize v0.8.1, keras v2.2.4 and tensorflow-gpu v1.12.0, Uniform Manifold Approximation and Projection (UMAP) v.0.5.2<br>Data simulations and Wilcoxon/Mann-Whitney U tests were performed in base R v3.6.1.<br>All code used for machine learning and power analysis is available at<br>https://github.com/SimonAB/DL-MIRS_Siria_et_al (doi:10.5281/zenodo.5996316).

For manuscripts utilizing custom algorithms or software that are central to the research but not yet described in published literature, software must be made available to editors and reviewers. We strongly encourage code deposition in a community repository (e.g. GitHub). See the Nature Portfolio guidelines for submitting code & software for further information.

Data

Policy information about availability of data

All manuscripts must include a data availability statement. This statement should provide the following information, where applicable:

- Accession codes, unique identifiers, or web links for publicly available datasets
- A description of any restrictions on data availability
- For clinical datasets or third party data, please ensure that the statement adheres to our policy

All relevant data are available from http://dx.doi.org/10.5525/gla.researchdata.1235 and Source Data File.

Field-specific reporting

Please select the one below that is the best fit for your research. If you are not sure, read the appropriate sections before making your selection.

☒ Life sciences ☐ Behavioural & social sciences ☐ Ecological, evolutionary & environmental sciences

For a reference copy of the document with all sections, see nature.com/documents/nr-reporting-summary-flat.pdf

Life sciences study design

All studies must disclose on these points even when the disclosure is negative.

Sample size	Sample size was chosen to maximize the number of spectra collected for each species/age group with time and resources available.
Data exclusions	Some mid-infrared spectra were excluded from the analysis if failed quality assessment as described in the manuscript. These exclusion criteria were pre-established.
Replication	Replication was ensured by collecting multiple samples with the same traits (e.g. species or age) in different laboratories. All attempts at replication were successful.
Randomization	Sample collection (e.g. collection of mosquito of given age/species) was conducted randomly from experimental cages/semifield systems. Samples (mosquitoes) were randomly allocated into experimental groups.
Blinding	Blinding was not relevant to the study as to perform machine learning analysis the training /test and validation set needed to be known.

Reporting for specific materials, systems and methods

We require information from authors about some types of materials, experimental systems and methods used in many studies. Here, indicate whether each material, system or method listed is relevant to your study. If you are not sure if a list item applies to your research, read the appropriate section before selecting a response.

Materials & experimental systems

n/a	Involved in the study	
☒	☐	Antibodies
☒	☐	Eukaryotic cell lines
☒	☐	Palaeontology and archaeology
☒	☐	Animals and other organisms
☒	☐	Human research participants
☒	☐	Clinical data
☒	☐	Dual use research of concern

Methods

n/a	Involved in the study	
☒	☐	ChIP-seq
☒	☐	Flow cytometry
☒	☐	MRI-based neuroimaging
